# Utstein-Style Template for Uniform Data Reporting of Acute Medical Response in Disasters

**DOI:** 10.1371/4f6cf3e8df15a

**Published:** 2012-03-23

**Authors:** Michel Debacker, Ives Hubloue, Erwin Dhondt, Gerald Rockenschaub, Anders Rüter, Tudor Codreanu, Kristi L. Koenig, Carl Schultz, Kobi Peleg, Pinchas Halpern, Samuel Stratton, Francesco Della Corte, Herman Delooz, Pier Luigi Ingrassia, Davide Colombo, Maaret Castrèn

**Affiliations:** Research Group on Emergency and Disaster Medicine, Vrije Universiteit Brussel, Belgium. Academy for Emergency Management and Disaster Medicine (EMDM Academy).; Research Group on Emergency and Disaster Medicine, Vrije Universiteit Brussel, Belgium; Academy for Emergency Management and Disaster Medicine (EMDM Academy); Research Group on Emergency and Disaster Medicine, Vrije Universiteit Brussel, Belgium. European Society for Emergency Medicine (EuSEM) Disaster Medicine Section.; World Health Organization (WHO) Regional office for Europe, Copenhagen, Denmark; Centre for Teaching and Research in Disaster Medicine and Traumatology, Linköping and Sophiahemmet University College, Stockholm, Sweden. European Society for Emergency Medicine (EuSEM) Disaster Medicine Section.; Bunbury and Busselton Hospitals, Western Australia and Dr. Gray's Hospital, Elgin, Scotland. European Master in Disaster Medicine (EMDM) Alumni Organization.; Center for Disaster Medical Sciences, University of California, Irvine, USA. World Association for Disaster and Emergency Medicine (WADEM).; Center for Disaster Medical Sciences, University of California, Irvine, USA.; National Center for Trauma and Emergency Medicine Research, the Gertner Institute for Health Policy and Epidemiology, Israel. World Association for Disaster and Emergency Medicine (WADEM).; Department of Emergency Medicine, Tel-Aviv Medical Center, Israel. European Society for Emergency Medicine (EuSEM) Disaster Medicine Section.; School of Public Health and David Geffen School of Medicine, University of California, Los Angeles. World Association for Disaster and Emergency Medicine (WADEM).; Center for Research and Education in Emergency and Disaster Medicine, Department of Experimental and Clinical Science, Novara, Italy. Academy for Emergency Management and Disaster Medicine (EMDM Academy).; Emeritus Professor, Catholic University Leuven, Belgium. European Society for Emergency Medicine (EuSEM) Disaster Medicine Section.; Center for Research and Education in Emergency and Disaster Medicine, Department of Experimental and Clinical Science, Novara, Italy. European Master in Disaster Medicine (EMDM) Alumni Organization.; European Master in Disaster Medicine (EMDM) Alumni Organization.; Department of Clinical Science and Education, Karolinska Insitutet, Stockholm, Sweden.

## Abstract

Background: In 2003, the Task Force on Quality Control of Disaster Management (WADEM) published guidelines for evaluation and research on health disaster management and recommended the development of a uniform data reporting tool. Standardized and complete reporting of data related to disaster medical response activities will facilitate the interpretation of results, comparisons between medical response systems and quality improvement in the management of disaster victims. Methods: Over a two-year period, a group of 16 experts in the fields of research, education, ethics and operational aspects of disaster medical management from 8 countries carried out a consensus process based on a modified Delphi method and Utstein-style technique. Results: The EMDM Academy Consensus Group produced an Utstein-style template for uniform data reporting of acute disaster medical response, including 15 data elements with indicators, that can be used for both research and quality improvement. Conclusion: It is anticipated that the Utstein-style template will enable better and more accurate completion of reports on disaster medical response and contribute to further scientific evidence and knowledge related to disaster medical management in order to optimize medical response system interventions and to improve outcomes of disaster victims.

## Introduction

A significant limitation in research studies on the disaster medical management is the lack of standards for collecting and reporting data^1^ ^4^. However, recent years have seen the publication of several guidelines for a common reporting structure^2^ ^5^ ^6^ ^7^. Nevertheless, the challenge of developing a rigorous and scientifically-based set of standardized data elements still remains. The availability of such data would enable researchers, educators and managers to study different aspects of the disaster medical response (DMR)^8^. To address this important issue, the Academy for Emergency Management and Disaster Medicine (EMDM Academy), an association comprised of research and education centres 9, initiated a project to establish a set of consensus-derived data elements using the well-documented Utstein-style method, including definitions and indicators for acute DMRs. Uniform Utstein-style reporting of data regarding out-of-hospital cardiac arrest, major trauma, and emergency medical dispatch, among others, has improved the international consensus on knowledge and practice in these areas^10^ ^11^ ^12^. In line with other already published Utstein-style templates, the purpose of implementing a uniform reporting system of essential data, with respect to DMR, is to take advantage of the reporting system’s ability to conduct research, evaluation, training, and practice and quality improvement programs^13^. Therefore, there is a need for a set of measurable indicators which should define the key descriptors (data elements) that have impact on DMR, as well as the methods of measurement 14 15. To date, the few metrics that exist have not yet been fully validated, nor have they universal acceptance 14 15 16. This project proposes a set of data elements and indicators that can be used to uniformly collect and report on the data required to perform research and education related to the medical response in disaster situations.

**Definition of Disaster**

A review of the literature reveals the challenge that researchers and professionals face in having to agree on a generally accepted definition or conceptual interpretation of “disaster”. Over the years, books and entire journal issues have been dedicated to answer the question: “What is a disaster” 17 18 19. Most of these definitions are valid in their respective context and reflect the authors’ opinions, as influenced by their disciplines or professional backgrounds^1^ ^20^ ^21^ ^22^ ^23^ ^24^.

The EMDM Academy Consensus Group – hereafter referred to as the Group – accepted from the outset that formulating a universally acceptable and all-encompassing definition of “disaster” was impossible. However, the Group was of the opinion that it was more important and feasible to identify, define and agree on the key characteristics that impact the DMR. This would allow for the development of a conceptual framework, which would better link these essential features. The Group also agreed that, from a DMR point of view, a disaster is an event in which the medical need exceeds the response capabilities in the affected area, mainly due to a large number and/or severity of injured or ill victims. This imbalance can be due to a quantitative and/or a qualitative shortage of resources (personnel and materials), but also to organizational or operational shortcomings. In this document, the word “disaster” is used as a synonym to “emergency” and “mass casualty incident/event”.

## Methods

**Inclusion/exclusion criteria**

Disaster management is comprised of four stages or phases: mitigation, preparedness, response and recovery. This project does not include the mitigation, preparedness or the recovery functions, although we recognize that these activities have a role in influencing the implementation of an adequate DMR system. The consensus process for this study only addressed the acute DMR phase where the involvement of the healthcare professionals is recognized as having the greatest immediate impact on the outcome of the disaster casualties. Similarly, although public health interventions and mental health support can be part of the acute medical response, this study will only focus on the acute management of physically injured or ill patients, from the scene of the disaster to definitive treatment, otherwise known as the disaster acute medical care response.

The consensus process was conducted using the combination of a modified Delphi technique and the Utstein-style method, which is a modified nominal group technique^25^ ^26^.

The Consensus Group was composed of 20 people, represented eight countries and consisted of experts in the fields of research, education, ethics, and operational aspects of disaster medical management. Taking advantage of the two-week annual training session of the European Master in Disaster Medicine (EMDM)^27^, the Group first met in Novara (Italy) in June 2009 for many of them are also EMDM faculty members. The Group members represent the following organisations: the Disaster Medicine Section of the European Society for Emergency Medicine, the World Association for Disaster and Emergency Medicine, the World Health Organization, and the Academy for Emergency Management and Disaster Medicine.

During the first meeting, the Consensus Group decided that the main use for the data collected during a disaster would be to perform research and investigations of the acute medical care response in mass casualty incidents. It was determined that other aspects of the health response, such as public health, mental health and mass fatalities, would represent future projects.

The Consensus Group also defined the main factors which impact the acute DMR, i.e. the number, type and severity of the ill/injured survivors, time factors, environment factors, and the DMR system.

Subsequently, the Consensus Group, composed of the 16 authors, met in Brussels in March 2010 and was divided into four discussion subgroups in order to identify a list of potential data elements and indicators that would characterize the four main impact factors, based on the current disaster medical management and operations literature.

The third step consisted of a virtual modified Delphi technique using the virtual portal of the EMDM Academy and coordinated by the group leader (MD). The 16 group members were requested to indicate which data elements and indicators they considered essential for research and the evaluation of the acute medical response in disaster situations. The responses and their justifications were analysed and only those data elements and indicators which had been selected by at least 80% of the group members remained in the list. The remaining data elements and indicators were re-distributed to the Group members for a second round with the goal of reaching full consensus. Lack of comment was deemed implied agreement.

A final meeting with the 16 authors was organized at the Utstein Abbey, on the island of Mosterøy, off the Coast of Stavanger, Norway (23-25 November 2010). Utstein meetings are characterized by strong international collaboration and sponsorship of scientific organizations, using a process of gathering a number of experts in an isolated intellectual environment that engage in well-facilitated discussions. The employed consensus method is the “Utstein rotations” format, a modified nominal group technique. After an introductory session, the group is divided into four discussion panels, each of which are chaired by a moderator (ED, GR, AR, MD), and allocated to a fixed “station”. Such a subgroup meeting was governed by the following procedural format: the moderator briefly introduced the data elements to be discussed; then each member of the subgroup was asked to express his/her viewpoint on the subject; subsequently, the subgroup formed a statement that was representative of the opinion of the subgroup as a whole; after 90 minutes, the members of the subgroup moved to another “station”; with each incoming group, the moderator stated the conclusions of the previous group and subsequently, each member of the new subgroup was asked to express his/her opinion; this procedure was repeated, so that each participant was given the opportunity to contribute to each topic. The moderators then presented the overall conclusions of each “station” to the whole group, so that the remaining disagreements could be more widely debated. The final consensus on each data element and its indicators was then considered ratified if there was a complete absence of “nay” votes and rejected if one or more “nay” votes were recorded. All selected indicators were considered core data, i.e. data that should be collected.

A writing meeting was thereafter organized in Brussels in April 2011, followed by several e-meetings, until the time of publication.

## Results

The data elements of the DMR including the indicators and metrics are listed in Table 1. The data regarding the pre-event settings and the disaster event description can be collected according to previous published guidelines, but should include at minimum the data listed in Tables 2 and 3 respectively.

**Figure:**
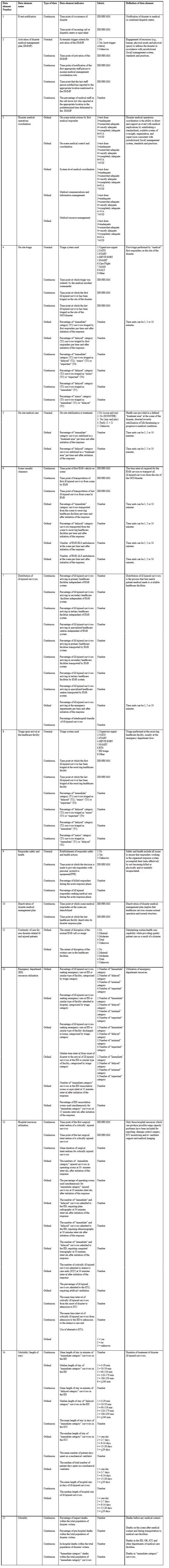
Table 1. Disaster Acute Medical Response data elements and indicators

**Table 2. Pre-event Setting**

**1. Population demographics**

- Population size

- Age disaggregated

- Gender disaggregated

- Vulnerable groups

- Crude Mortality Rate (CMR)

- Under-5 Mortality Rate (U5MR)

- Endemic diseases – health profiles

**3. Healthcare System (including capacities)**

- Dispatch system

- EMS System

- Healthcare Facilities (primary, secondary, tertiary and specialty centers)

**4. Disaster Medical Response System**

- Medical Incident Management system including the different tiers (lines of authority)

- Criteria for activation of the disaster medical management plan (DMMP)

- Medical operations plan

- Pre-hospital care
- Medical care

- Concept of operations

- Event recognition/initial notification
- Activation/mobilization
- Response
- Demobilization

- Medical information management

- within own organization
- scene and medical command
- to other agencies
- to authorities
- to public
- to media
- communication resources and dissemination system

- Medical resources management

- Personnel
- Supplies and equipment
- TransportationTechnical support

**Table 3. Event Description**

**1. Type of Event**

- Hazard type

- Warning

- Disaster scene (one or multiple)

- Duration (static or dynamic)

- Contagious or not

**2. Impact intensity/magnitude**

- Intensity scale

**3. Impacted Area**

- Location

- Surface

**4. Subsequent related events**

- Hazard type

- Warning

- Disaster scene (one or multiple)

- Duration (static or dynamic)

- Contagious or not

**5. Time of onset**

- DD/MM/YY HH:MM

- Day of the week

- Holiday

**6. Environment**

- Weather (temperature, humidity, precipitation type and amount, wind speed and direction)

- Height

**7. Overall damage severity**

- Score

**8. Damage to infrastructure and life-sustaining services**

- Potable water

- Sanitation

- Power (electricity)

- Energy relative to life sustainment

- Transportation

- Communication

- Fuel

**9. Damage to healthcare system**

- EMS System

- Healthcare Facilities (primary, secondary, tertiary) and Specialized Facilities

- Access to healthcare services

**10. Casualties**

- Age group (0-1 y, 1-17 y, 18-65 y, >65 y)

- Primary casualties

- Secondary casualties

- Casualty rate (number of ill/injured survivors / population exposed)

- Impacted (affected) population

- Severity of injuries (urgent versus non-urgent, hospitalized versus non-hospitalized, the ratio of immediate (T1) + delayed (T2) category survivors to the minimal (T3) category survivors

**Event notification**

In some events (transportation accidents, explosions) the impact will stretch or overwhelm the baseline of the actual healthcare system, however, this may not be readily apparent in other events, such as heat waves or epidemics. In addition, the initial recognition of the magnitude of the impact on the health system may be unclear even in obvious but widespread disasters^28^. Consequently, the event identification and notification could be immediate or delayed. According to the Group this can be measured by determining the interval between the time points of the disaster’s occurrence and the incoming call at the appropriate dispatch centre or equivalent in order to activate the medical response.

**Activation of the disaster medical management plan (DMMP)**

The activation of the DMMP and the subsequent notification of the appropriate medical functions should occur almost simultaneously. Notification consists of any process in which the appropriate medical functions and services are informed of a disaster situation that may require a response from those notified.

Any event that can cause a severe impact on routine healthcare services should be considered the threshold for the activation of the DMMP at the appropriate management tier in order to maximize the medical resources. Several trigger criteria for the activation of the DMMP, generally based on the total number of victims compared with system resources, the number of serious ill or injured survivors and/or loss of critical healthcare assets, are already in use^28^.

The Group proposes the following indicators to measure the effectiveness of the activation process of the DMMP:

1. the promulgation or not of trigger criteria for activating the DMMP;
2. the time point of activation of the DMMP;
3. the time point of notification of the first appropriate staff person to assume medical management coordination role;
4. the time point that the last staff person notified has reported to the appropriate location mentioned in the DMMP; and
5. the percentage of medical staff on the call-down list who reported to the appropriate locations in the predetermined time delineated in the DMMP.

**Disaster medical operations coordination**

Disaster management is the universal term meant to describe the structures and processes utilised to mitigate, prepare for, respond to and recover from, a disaster.

Disaster medical operations consist of all medical and non-medical actions required to achieve the response objectives following the activation of the DMMP.

The diverse organizations involved in the disaster response do not routinely function together, thus creating challenges in communication and coordination within the response system. Furthermore, disasters often involve activities across multiple levels of government^28^ ^29^.

Disaster medical operations coordination is the ability to respond to an event with health impacts by establishing a structure of supervision and organization consistent with the jurisdictional disaster management system and disaster medical management concept, principles and standards^30^ ^31^.

To perform the activities necessary to address the immediate and short-term health effects of a disaster, the health discipline must fall back on an integrated medical management system composed of an operating structure and response processes. The operating structure involves the division of tasks, including roles, responsibilities and authorities, as well as the coordination of diverse medical and non-medical operational assets. It is essential that these assets function together effectively towards minimizing mortality and morbidity of the disaster survivors. Rescue, decontamination, triage, stabilization, evacuation, and definitive treatment of disaster survivors, performed by all the involved operational assets, require integration through individual, mono- and/or multidisciplinary disaster management or coordination systems^29^ ^31^ ^32^.

The response management activities include ad-hoc problem-solving, planning, decision making, communication, monitoring and evaluation^33^.

The main, ongoing, activities of medical operation coordination for an effective response include:

- maintaining situational awareness by continuous data collection and analysis. The data collected relates to the health impact, the health needs and available resources in and outside the affected area and the anticipated medical development of the disaster;

- medical communications and information management by establishing communication pathways between coordinating bodies and operational organizations, providing information to all stakeholders involved in the response, issuing progress reports at regular intervals, providing public information and interacting with the media;

- medical resource management by maintaining an inventory of available health resources (human and material), matching available health resources with defined needs and procuring and allocating health resources for unmet needs;

- management of the medical response by exercising authority to direct and control all aspects of the medical response by reaching consensus concerning goals and objectives of the response (medical response strategy) and the priorities for action (medical action plan). It also includes continuous monitoring in order to detect new problems and evaluate the effectiveness of the response activities and interventions, and providing quality assurance and control^1^ ^31^.

To date, there has been limited research to identify, develop and validate indicators for measuring medical response management^1^ ^8^ ^34^ ^35^ ^36^ ^37^ ^37^. In particular, the coordination process in the event of a disaster, due to the very nature of the operational and ethical constraints, has been the most challenging.

To fill this gap, the group developed a qualitative “Medical Operation Coordination Scale” in order to collect the required information on the performance of acute response actions or interventions in which coordination must be performed in a specific order to be effective (Appendix 1). Scoring of the effectiveness of the interventions is based on the method proposed by Parker et al^208^. For assessment purposes, “effectiveness” means that the action or intervention in question is both sufficient and timely enough so that the task to which it pertains can be accomplished. The scale is designed to provide an adequate number of categories to capture variation among ratings and allows for non-ratings through the “non applicable” and “not able to determine” response options, when appropriate. The scoring methodology and possible weighing of items on the Medical Operation Coordination Scale must await further validation through pilot-testing and field-testing.

The data required for measuring the effectiveness of the coordination of the DMR are focused on the essential health functions and management processes necessary to address the immediate and short-term health effects of a disaster at each level of the response. These include:

- On-scene initial actions including the assessment of the preliminary health effects;

- On-scene medical control and coordination including the development of a medical action plan;

- System-level medical coordination;

- Medical communication and information management; and

- Medical resource management.

Measuring the timeliness is fundamental because decision-making involving the mobilization of staff and other resources and establishing operational facilities and coordination centres are critical, ultimately affecting the time interval in which the survivors will receive their medical treatment.

In addition to speed, scale is also important. For dispensing medical care, throughput metrics enable calculation of the overall rate at which the ill/injured survivors will receive treatment.

Staff call-down is the ability to contact and mobilize staff to perform disaster response functions. For call-down, estimation metrics enable a count of the number of people who could be mobilized to medical management functions. Since the goal is to recruit staff for duty in a disaster, the calls must be conducted in a timely fashion, and the calls must reach all the necessary health personnel. In an actual disaster, the result should be that the response assets could be gathered at the time they are required. Consequently the “indicators” test the validity of the response system call-down lists and their ability to contact those staff in a timely matter: it estimates the percentage of staff who are able to report for duty to a given location at a certain time^38^.

The Group endorses the indicators proposed by Nelson et al^38^:

1. call-down completion time (time duration required to call all staff on the call-down list);
2. acknowledgement time (time duration required to receive response from staff confirming receipt of the call-down message, regardless of ability to assemble);
3. acknowledgement percentage (the proportion of staff on the call-down list who confirmed receipt of the call-down message, regardless of ability to assemble); and
4. the assembly percentage (the proportion of staff on the call-down list who reported at a designated site by a predetermined target time).

***On-site triage***

From a medical point of view, a disaster is characterized by the disproportion between the increased demand for medical response capacity and the actual medical resources available in order to manage the ill/injured survivors. This disparity can be due to a quantitative and/or qualitative shortage of human or physical resources as well as management problems. Therefore, it is imperative to set medical and organizational priorities. Due to prioritisation, not each individual victim will receive the optimal care immediately, and a certain selection of casualties is inevitable^39^.

Triage is often considered in narrow terms or as an isolated process, such as the categorization of casualties based on injury severity or the prioritization of patient treatment and/or transport^39^ ^40^ ^41^ ^42^. Triage is a management tool meant to achieve the primary objectives of disaster medical management. It seeks to rapidly provide the greatest benefit for the largest number of casualties in order to minimize mortality, morbidity and indirect effects within the affected population and to re-establish as soon as possible the routine level of medical care. It also renders the delivery of medical care to an overwhelmed health system into a manageable task 39 41 43 34 35 36 37 38 49.

The main factors that influence the triage process include the type and severity of the injuries/illnesses, the number of ill/injured survivors, the spatiotemporal distribution of the survivors, and the DMR system^42^ ^50^ ^51^ ^52^ ^53^ ^54^ ^55^.

1. The type and the severity of the injuries/illnesses according to the nature of the disaster: wounded, burned, blasted, intoxicated or contaminated victims, polytrauma patients, and casualties with combined injuries. The most important patients to identify are those critically ill or injured who will benefit immediately from the provision of resuscitative stabilization. Triage tools should identify which survivors are in need of immediate treatment as opposed to those whose treatment can be delayed and those who do not require medical treatment^56^.

2. The number of victims: the great majority of immediate disaster survivors are not critically injured or ill, as most lethal injuries or exposures kill immediately^4^ ^52^ ^57^ ^58^. There is no simple method by which critically ill/injured survivors can be identified on the site of the disaster. This creates the potential for overtriaging to occur. However, in a recent study using the START system, 100% of “immediate category” survivors were identified in an actual disaster^59^. This is where triage can make its greatest impact in the acute medical response in managing the number of victims. The outcome of these “immediate category” survivors is a good indicator of the effectiveness of medical care in disasters^52^ ^60^ ^61^.

3. The spatiotemporal distribution of the casualties:

- the spatial distribution of victims depends on the extent of the disaster area: the greater the distances, the more difficult the rescue and evacuation of the victims;
- the temporal distribution: the acute medical response will be different if it experiences a massive influx of casualties within a short period of time versus a steady flow of victims 62 63.

4. The DMR system: triage management requires a tiered operational DMR system, which will define the extent and availability of resources^42^ ^51^ ^64^. This requires effective coordination and information management within the entire DMR system^41^ ^43^ ^55^ ^61^ ^63^ ^65^.

It is well known that a short interval between the initial injury and definitive medical treatment offers the best chance of survival, shortens the period of convalescence and rehabilitation, and decreases functional disability^66^. Therefore, one of the most important tasks of the health sector is the setting-up of a well-organized evacuation system for the victims from the scene of the disaster to the appropriate healthcare facilities. Quick and accurate triage of the casualties at each level of the medical care chain is the cornerstone of the efficacy of such a system. At the scene of the disaster, triage is applied to determine mainly evacuation priorities. Appropriate distribution of patients among the available healthcare facilities reduces the burden to a manageable level and allows for the efficient use of resources. The triage process continues in hospitals but will be based on treatment priorities such as surgery and intensive care.

Consequently, the triage process must be seen in a wider context and is comprised of the following elements:

1. Rapid evaluation of all disaster victims;
2. Assessment of the nature and severity of the injuries and their consequences on the vital functions of the casualties;
3. Categorization of the casualties;
4. Stabilization and conditioning for transport;
5. Distribution and evacuation of the casualties;
6. Admission, if appropriate, to healthcare facilities for definitive care.

By fulfilling the aforementioned recommendations, disaster response activities will take place in a sequential and comprehensive fashion which minimizes patient care burden at each subsequent level of intervention and reduces the overall need for rationing care. As such, triage is one of the medical surge tools of the DMR system^42^ ^67^.

Triage is a progressive process. A primary (preliminary, early, swift, sweeping) survey that quickly reviews all victims should be in place. This survey would essentially determine the number of casualties, type and severity of injuries, and would identify those critically injured individuals who will immediately benefit from basic resuscitative procedures. Triage becomes more thorough and accurate, as the ill/injured survivors move along the lines of medical care and as the disproportion between the needs and available resources lessen^68^ ^69^. Triage is also a continuous process throughout the medical evacuation chain and is applied for as long as the disproportion between casualty demands and medical resources is ongoing. Primary triage is the first stage of patient triage, which usually occurs in the pre-hospital setting, but can occur in the emergency department for self-referred victims. Secondary triage is the re-evaluation of the patient’s condition after initial medical care and may occur at the scene, at an alternative medical treatment site, or at the hospital. Tertiary triage is the re-evaluation of a patient’s condition after further medical care and is ongoing during the patient’s stay in a healthcare facility. Thus, patients are continually reassessed at all levels of care throughout the entire journey of care^43^ ^52^ ^60^ ^70 71^ ^72^ ^71^ . Triage is also a dynamic process. To perform accurate triage, the following factors need to be considered: the nature, extent and severity of the injuries, the existence of associated or combined injuries, the mechanism of injuries, the prognosis, the change in the patient’s condition, the effectiveness of the treatment, the age of the patient, the number of patients, and cultural and religious factors^44^ ^46^ ^60^ ^73^ ^74^. The triage decision process is additionally complex in disasters when following factors must be taken into consideration: effects of the disaster, location and accessibility of casualties, hostile environmental conditions, delay in treatment, limited resources in manpower, material, transportation means, distances to hospitals and treatment capacities of the receiving healthcare facilities 43 60 73 75 76 76.

Being a progressive, continuous and dynamic and process, the triage category allocated to each injured/ill is adjusted as appropriate, and no triage decision should be considered final for as long as the disaster situation is ongoing^69^.

The accuracy of triage decisions may be an important factor in the effectiveness of the medical response and erroneous decisions may potentially compromise the clinical outcomes^52^ ^61^. Undertriaging in disasters is defined as the inappropriate assignment of “immediate category” survivors to receive delayed or minimal care. Overtriaging in disasters is defined as the inappropriate assignment of “delayed category” and “minimal category” survivors to receive immediate care. To date, only one triage system (START triage) has been retrospectively validated under disaster operational conditions^59^.

Further research is necessary to:

1. establish whether existing disaster triage systems are of any value as a management tool in disasters, providing optimal resource utilization and improved patient’s outcome with respect to mortality and morbidity^56^ ^77^ ^78^ ^79^ ^80^ ^81^ ^82^;
2. define a universally accepted measure of triage accuracy^78^ ^79^;
3. establish whether the criteria used to sort ill/injured survivors into categories are clinically meaningful and are adequately predictive of survivability^42^ ^81^;
4. study the performance characteristics of all the various triage tools^56^ ^81^ ^82^; and
5. to address non-traumatic disaster scenarios^80^ ^82^ ^83^ ^84^.

The Group proposes the following indicators of the triage process:

1. the triage system used;
2. the time point at which triage was ordered by the medical incident commander;
3. the time point at which the first and last ill/injured has been triaged survivor on the site of the disaster;
4. the percentage of “immediate” category (T1) and “delayed” category (T2) survivors triaged by first responders per time unit (1 minute, 5 or 10 minutes interval) after initiation of the response, or put differently, the total distribution of “immediate” and “delayed” category survivors with respect to the time they are triaged. This indicator would indicate how well the responders identify those who need urgent medical assistance; and
5. the rate of undertriage and overtriage.

**On-site medical care**

The response to a disaster is time-sensitive and requires rapid intervention by healthcare providers in order to minimise death and morbidity^85^ ^86^ ^87^.

Activities at the disaster scene include the collection, medical treatment and transport of the ill/injured survivors. The collection process includes victim location, primary triage and retrieval of the ill/injured survivors from the direct hazard impact area(s) and transfer into the formal system for evacuation to definitive care. This transfer may involve either holding the victims in one or more areas for on-site medical care or direct transfer to healthcare facilities. The medical treatment and transport process is comprised of:

- secondary triage,
- on-scene treatment and release of minimally ill/injured individuals,
- advanced stabilization, distribution and transport to healthcare facilities of the more seriously ill/injured survivors^87^.

Data relating to the triage and distribution/transportation of ill/injured survivors are handled as separate data elements.

For all victims, it is important to weigh the need for pre-hospital interventions against the need for prompt transport to the healthcare facility^88^. A pre-hospital time interval of greater than 60 minutes (the so-called “golden hour”^89^ is traditionally associated with a significant increase in the risk of death for severely injured patients^90^. However, this 60 minute cut-off point has no scientific basis^88^. Recent studies have concluded that there are no “golden” timelines^91^ and that there was no significant association between time and mortality for any EMS time interval (activation, response, on-scene, transport and total pre-hospital interval)^92^ ^93^ ^94^.

Furthermore, the pre-hospital care of trauma patients is surrounded by much controversy^88^ ^95^ ^96^. There is growing evidence questioning the benefit of specific advanced interventions for the pre-hospital management of severely injured patients^94^ ^97^ ^98^ ^99^ ^100^. Advanced interventions may reduce morbidity or mortality in disasters with a large number of ill/injured survivors, in disasters possessing environmental obstacles, and in situations where the capacity of the healthcare system is temporarily overwhelmed, the means of evacuation are insufficient or the time for evacuation is prolonged^74^ ^76^ ^88^ ^101^. The following advanced interventions may influence victim outcome:

- maintaining an open airway,
- decompressing a tension pneumothorax,
- drainage of a hemo-pneumothorax,
- controlling external exsanguinating haemorrhage,
- fluid resuscitation,
- spinal immobilization
- analgesia, including the splinting of major limb injuries^61^ ^74^.

Unfortunately, the implementation of advanced interventions in disaster situations or even in pre-hospital trauma care in rural areas has still to be scientifically validated^61^ ^88^. Furthermore, we do not know whether the health outcome of the disaster survivors is improved if the response is led by trained doctors as compared to trained emergency medical technicians^102^.

The Group proposes the following indicator to measure the effectiveness of the on-site medical care process:

1. the execution or not of on-site stabilization or treatment; and
2. the percentage of immediate and delayed survivors stabilized in an on-scene “treatment area” per time unit (1minute, 5 or 10 minutes interval) after initiation of the response, or put differently, the total distribution of “immediate” and “delayed” category survivors with respect to the time they are stabilized on-scene.

**Scene casualty clearance**

There are several approaches for managing a disaster scene. Some authors associate scene organization with the goal of rapid transport and limited pre-hospital medical interventions (“scoop-and-run”). Others emphasize more extensive field medical interventions prior to transport (“stay-and-play”). The differences of opinion on this controversial issue (of stay-and-play versus scoop-and-run) could perhaps be harmonized by a “scoop-treat-and-run (SCOOTER)” system. This means that evacuation of the survivors should begin as soon as possible and that stabilization measures should be performed once the patient transport to the healthcare facility has started^61^ ^101^. It is likely that in a rural area the patient transfer may take longer. Therefore, on-scene stabilization may be advisable prior to transport to the appropriate hospital^74^ ^76^ ^101^.

The Group proposes the following indicators to measure scene casualty clearance:

1. the time point of the first EMS ambulance on scene;
2. the time interval between transport of the first and last survivor from the scene by EMS ambulances;
3. . the percentage of “immediate” and “delayed” category survivors transported from the scene to receiving healthcare facilities per time unit (1 minute, 5 or 10 minutes interval) after initiation of the response; and
4. the number of EMS (BLS and ALS) transportation means at the scene per time unit (1 minute, 5 or 10 minutes interval) after initiation of the response.

***Distribution of ill/injured survivors***

Distribution of ill/injured survivors of disasters consists of best matching their medical needs to the available facilities, specialities, and admission capacities of healthcare facilities and the transportation modalities that accomplish the movement of victims to those healthcare facilities^85^.

Transportation of ill/injured survivors should start as soon as possible, according to the severity of illnesses or injuries, readiness of victims to evacuate and the availability of transportation means. Transportation assets have to match the needs of victims. Adequately trained and equipped healthcare providers should be available to care for seriously ill/injured survivors, while minor cases should be transported by any means possible, as long as they are approved by the officer in charge of the site^85^ ^101^.

Research observations indicate that a substantial number of disaster survivors, including severely injured patients, are transported to healthcare facilities by means other than an ambulance^102^ ^103^ ^104^ ^105^ ^106^ ^107^. In spite of this, research does not show whether disaster survivors benefit or suffer from rapid non-EMS transport compared with perhaps a more delayed but dedicated EMS ambulance transport.

Many studies on disasters have shown that it is common to witness a disproportionate number of survivors being transported from the scene to the closest healthcare facility^57^ ^103^ ^104^ ^105^ ^108^. In addition, self-transported survivors are also expected to present to the closest or most familiar healthcare facility^61^ ^102^.

Disasters can occur anywhere, and can affect metropolitan areas, containing one or more level-1 trauma centres, to remote areas, that do not have access to a nearby healthcare facility or to a fully equipped hospital^109^. These remote areas may have limited EMS capabilities, which further complicate the casualty disposition process^106^ ^110^. During incidents in which the needs of the ill/injured survivors outweigh the definitive care capacities of the receiving healthcare facility or when a prolonged, ongoing patient flow is expected, one healthcare facility can function as an “evacuation hospital”. This would involve receiving and stabilizing the disaster survivors and secondary distribution of all or part of those patients requiring further care to other facilities, depending on the patient load and local treatment capacity^62^ ^101^ ^109^ ^111^ ^112^ ^113^.

Maldistribution of ill/injured survivors in disasters is a recurring problem, as it overwhelms individual healthcare facilities and may not ensure optimal medical care^103^ ^104^ ^108^ ^114^ ^115^ ^116^ ^117^ ^118^ ^119^. The standard doctrine stipulates that victims should be transported to the most appropriate facilities. Several factors must be considered regarding the distribution and optimal use of hospital resources including the following:

- number and flow of ill/injured survivors,
- injury types and severity,
- evaluation of the needs of the ill/injured survivors,
- individual capacities and capabilities of the receiving healthcare facilities,
- distance to area healthcare facilities, and
- evacuation capacity of the response system^52^ ^85^ ^101^ ^109^ ^119^.

In a study of 33 terror-related MCIs, Einav et al showed that the majority of all survivors and patients in urgent care were evacuated to the nearest hospitals and not necessarily to trauma centres, even in large urban regions^112^. They suggest that critically and mortally ill/injured survivors should be immediately evacuated to the nearest healthcare facility for primary resuscitation and stabilization (evacuation hospital concept), and that all other casualties should be evacuated to all other healthcare facilities in the area to avoid overwhelming any one hospital^112^. Other authors disagree with the concept of transporting the critically injured victims to the nearest hospital as it contradicts demonstrated improved outcomes achieved by trauma centres^119^ ^120^ ^121^ ^122^ ^123^.

Overcrowding or underutilisation of the available healthcare facilities can occur if patient distribution is not judiciously planned and executed^119^ ^124^. Effective coordination and communications are of critical importance in the distribution of disaster victims^61^ ^101^ ^102^ ^109^ ^112^.

The Group proposes the following indicators for assessing the distribution process of disaster victims:

1. the percentage of ill/injured survivors arriving to primary, secondary, tertiary and specialized healthcare facilities transported by the EMS system;
2. the percentage of ill/injured survivors arriving in primary, secondary, tertiary and specialized healthcare facilities independent of the EMS system;
3. the percentage of ill/injured survivors arriving at emergency departments (or similar facility) per time unit (1 minute, 5 or 10 minutes interval) after initiation of the response; and
4. the percentage of inter-hospital transfer of ill/injured survivors.

**Triage at the healthcare facility**

Pre-hospital triage is not guaranteed, considering that disaster survivors can arrive to healthcare facilities via a number of transportation modes^125^ ^126^. Currently used emergency department triage scales, aiming to optimize the evaluation and treatment time of patients, according to the severity of their medical condition, may not be adequate for characterizing the nature and severity of the disaster surge^127^ ^128^. There is insufficient scientific evidence which positively correlates emergency department triage scales with predicted outcomes, and the same applies to disaster triage tools^80^ ^129^ ^130^.

Severely injured patients are not necessarily the first to arrive and may continue to arrive to the hospital for an extended, and often unknown, period of time^131^.

Some authors suggest that the sorting of incoming patients should be based both on the urgency of the treatment need and the anticipated utilization of important but scarce resources (resuscitation rooms, operating rooms, ICUs, medical imaging facilities). They proposed a simplified triage scheme with only 2 categories, i.e., all patients needing immediate interventions will be selected as priority, while all others will be assigned to the delayed care category, so long as their illnesses/injuries do not progress^43^ ^130^ ^131^ ^132^ ^133^.

The Group proposes the following indicators for the emergency department triage process:

1. the triage system used;
2. the time point at which the first and last ill/injured survivor has been triaged at the emergency department of the receiving healthcare facility; and
3. the undertriage rate and the overtriage rate.

**Responder safety and health**

Safety and health include all measures that ensure responders working in the organized response system can accomplish their tasks effectively without experiencing any physical or mental harm (130). First responders and hospital staff may be exposed to various environmental hazards during response operations, making them a significant liability to other victims and staff^134^ ^135^ ^136^ ^137^ ^138^. Furthermore, reports indicate that employees are less willing to report to duty during biological, chemical or radiation events, if they perceive a high degree of risk to themselves or their families^139^.

The Group proposes the following indicators that concern the safety and health of first responders and hospital staff:

1. the establishment of actions for safety and health;
2. the time point at which the decision is made to provide responders with personal protective equipment;
3. the percentage of killed and ill/injured responders during the acute response phase; and
4. the percentage of ill/injured responders seeking medical care during the acute response phase.

**Deactivation of operational plan.**

Demobilization refers to activities that focus on the rapid return of the stood-down medical response resources to their normal function^85^. Demobilization of individual assets may occur at widely varying times, as increased workload of individual facilities may last several days^28^. Other important activities of the demobilization process, besides re-establishment of normal EMS operations and the rescheduling of regular staffing and normal activities of the healthcare facility, include addressing staff rehabilitation and health concerns, completing and securing incident documentation, conducting incident debriefing and review, and performance evaluation^130^.

When studying the potential benefit of disaster response systems, the cost of providing a response, measured in terms of time that health professionals are absent from their usual duties, may be an important consideration.

Therefore, the Group proposes as indicators:

1. the time point at which scene medical responders at the scene are demobilized; and
2. the time point at which the last healthcare facility deactivates its disaster response plan.

**Continuity of care for non-disaster-related ill and injured patients**

It is likely that due to the increased load of disaster victims, a disruption of the normal activities of the pre-hospital and hospital services will occur^134^ ^140^. Therefore, the utilization of resources must be balanced with the need to maintain enough capacity to meet other local non-incident-related demands for medical care^61^.

The Group proposes the following indicators for the measurement of the continuity of care for non-disaster-related ill and injured patients:

1. the extent of disruption of the normal EMS call coverage; and
2. the extent of the disruption of routine care in healthcare facilities.

**Emergency department (ED) resource utilization**

As the link between pre-hospital medical operations and in-hospital definitive treatment, emergency departments hold an important position in the care process of ill/injured disaster survivors^127^. Available data suggests that emergency departments are the access points for health care in sudden impact disasters^4^ ^116^ ^141^ ^142^, and that the majority of survivors are low-acuity patients who are discharged from the ED the same day^4^ ^62^ ^102^ ^104^ ^124^ ^143^.

A hospital’s first notification of a disaster may often come with the first arriving victims, rather than from authorities in the field. Moreover, immediate information and updates about incoming victims are often insufficient or lacking^102^ ^103^ ^144^. The time it takes the first ill/injured survivor to arrive to the ED in a sudden impact disaster varies and can range from a few minutes to 30 minutes^102^ ^104^ ^124^ ^143^. In the majority of sudden impact disasters, the first peak of presentations occurs between 60 and 90 minutes, and most patients will have presented within 2 to 4 hours after the impact^67^ ^143^ ^145^ ^146^. In other sudden impact disasters like earthquakes, however, it may take several days before the ED volume returns to normal^140^ ^147^ ^148^.

The time interval from the onset of a disaster to the arrival of the first ill/injured survivor helps define the speed with which such incidents impact the EDs^116^. Factors likely to affect this time interval include hospital proximity, the ability of ill/injured survivors to transport themselves or be transported to EDs independent of the EMS system, and the prevalent traffic conditions^102^ ^116^ ^149^. The time of arrival of the last ill/injured survivor to the ED helps define the duration of the impact on the EDs^116^. Factors likely to influence this time interval are complex and include the hazard characteristics that affect the timeliness of search and rescue or evacuation, the number of victims, the pre-hospital response capacities, the effectiveness of casualty distribution and hospital proximity^116^. Appropriate ED response planning takes into account the time delay between the hazard impact and the first patient presentation, as well as the duration of patient influx^116^.

The “Emergency Department Time Interval” has been defined as the time lapse between ED triage to the time point of actual admission to the hospital^150^ ^116^. There is evidence suggesting that longer delays between the transfers from ED to ICU are linked to an increased mortality rate^151^.

Another important parameter of the health facility response is the rate at which ill/injured survivors arrive and use available resources. This rate is neither constant nor linear^67^ ^104^ ^116^ ^131^ ^152^ ^153^ ^154^). The ED influx of ill/injured survivors is not uniformly distributed over the duration of the medical response and treatment areas, such as ED, OR and ICU^67^. Estimations of surge by averaging patient numbers over the duration of the incident will underestimate the actual maximum surge rate^67^.

It is recommended to institute a unidirectional patient flow so that patient movement is uninterrupted, orderly and traceable. Patients will not be readmitted to the ED as their bed will likely be occupied by the next incoming casualty^29^ ^101^ ^132^ ^155^.

Another indicator of ED utilization is the critical surge to capacity ratio (ratio of “immediate category” patients to available resuscitation bays/rooms and teams), as it seems to have an impact on the mortality of “immediate category” injured survivors and as it has the potential to standardize the assessment of the ED capacity across disasters of varying size^67^ ^155^ ^156^ ^157^ ^158^. The increase of ED utilization or surge capacity in disasters is assessed by evaluating the ED treatment capacity in routine situations and after activation of the hospital disaster plan^159^.

The Group proposes the following indicators for the measuring of the utilization rate of emergency department resources:

1. the percentage of ill/injured survivors seeking emergency care at an ED or similar type of facility, categorized by triage category;
2. the percentage of ill/injured survivors seeking emergency care at an ED or similar type of facility admitted to the hospital, categorized by triage category;
3. the percentage of ill/injured survivors seeking emergency care at an ED or similar type of facility discharged home, categorized by triage category;
4. the median time interval from the onset of disaster to the arrival of ill/injured survivors at the ED or similar type of facility, categorized by triage category;
5. the number of “immediate category” survivors in the ED resuscitation rooms or equivalent at 15 minutes interval after initiation of the response;
6. the percentage of ED resuscitation rooms used simultaneously for “immediate category” survivors at 15 minutes intervals after initiation of the response; and
7. critical surge to capacity ratio.

***Hospital resources utilization***

Data concerning the utilization rate of operation rooms (OR), medical imaging, and intensive care units (ICU) should be collected, as these resources may be rate-limiting factors in the management of disaster survivors^160^.

The number of operating rooms may be a limiting factor in a high casualty load^114^. Current protocols advocate that only those casualties with life-threatening surgical conditions should be transferred to the operating rooms during the initial phase of victim influx^67^ ^125^ ^161^) and that only damage control resuscitation and surgery is performed at this stage 67 161 162 163. The Group proposes the following indicators for measuring the utilization rate of the operating rooms:

1. the time point of the first and last surgical intervention of an “immediate category” injured survivor;
2. the numbers of “immediate category” injured survivors in operating rooms at 30 minutes intervals, after initiation of the response;
3. the percentage of operating rooms used simultaneously for “immediate category” injured survivors at 30 minutes intervals, after initiation of the response; and
4. the mean duration of the surgical interventions for “immediate category” injured survivors.

Published reports from terrorist bombings noted that between 45% and 81% of injured survivors required plain radiology, 78% to 90% required computed tomography, and 12% to 38% required ultrasound sonography^67^ ^104^ ^164^ ^165^ ^166^ ^167^. Fast and accurate imaging including radiography, computed tomography and ultrasonography are used to help determine which ill/injured patient will be triaged to immediate surgery or an ICU^168^. With the exception of ultrasonography, which has proven to be an effective screening tool in disasters^132^ ^161^ ^169^ ^170^, no uniform policies exist with respect to the use of radiography and computed tomography in the initial phase of ill/injured survivors influx^117^ ^131^ ^132^ ^161^ ^168^ ^170^.

The Group proposes the following indicators for measuring the utilization rate of medical imaging modalities: the number of “immediate” and “delayed” survivors admitted to the ED, requiring medical imaging at 30 minutes intervals after initiation of the response, categorized by imaging technique (plain radiography, computed tomography, ultrasonography).

Disasters such as major chemical incidents, earthquakes, tsunamis, terrorist bombings and industrial explosions or pandemics can generate a large number of victims with critical clinical conditions requiring intensive care^171^ ^172^ ^173^ ^174^ ^175^. The intensive care surge demands may need to be sustained from days to weeks, in comparison to emergency department response, that typically only last a limited number of hours to days^67^ ^155^ ^171^. It is likely that the need for a rapid expansion of the ICU capacity in a mass casualty will prove extremely challenging^171^ ^177^ ^178^. Due to insufficient critical care staff, medical equipment and ICU space, the provision of a timely, high-quality critical care to a surge of critically ill or injured patients will prove difficult in many countries^178^ ^179^ ^180^ ^181^. In addition, ventilator availability may be another potential bottleneck as there are few acceptable alternatives^152^ ^181^ ^183^ ^182^. The Group proposes the following indicators for measuring the utilization rate of ICUs:

1. the number of critically ill/injured survivors admitted into an ICU at 30 minutes intervals after initiation of the response;
2. the percentage of ill/injured survivors admitted to the ICU, requiring artificial ventilation;
3. the mean time interval from the start of the event to admission into the ICU;
4. the mean time interval between admission to the ED to admission to the ICU; and
5. the use of alternative ICUs.

**Outcome indicators**

Currently, only one study provides evidence of a poorer outcome for surge patients originating from actual disasters than for routine trauma casualties that typically present one case at a time^184^.

Disasters, depending on their type and magnitude, result in various levels of morbidity and mortality. Most healthcare providers agree that the aim of the DMR is to “save as many lives as possible”, but no consensus exists as to what exactly constitutes a disaster or on how to deliver medical care to achieve this goal^71^ ^72^ ^101^ ^185^ ^186^ ^187^ ^188^ ^189^. Further research is required to investigate the ill/injured survivors’ outcomes with respect to DMR both in the pre-hospital and in-hospital phase. Factors such as the type of hazard or injury mechanism, field and ED triage, ill/injured survivors’ (peak) loads, speed of evacuation, distribution of victims to healthcare facilities, alterations in care, organizational issues, and education and training in disaster medical management of healthcare providers should be investigated^80^ ^101^ ^114^ ^156^ ^184^ ^189^ ^190^ ^191^.

We propose the following indicators for measuring morbidity:

1. the mean length of stay in minutes of “immediate” category survivors in the ED;
2. the median length of stay of “immediate” category survivors in the ED;
3. the mean length of stay in minutes of “delayed” category survivors in the ED;
4. the median length of stay of “delayed” category survivors in the ED;
5. the mean length of stay in days of “immediate” category survivors in the ICU;
6. the median length of stay of “immediate” category survivors in the ICU;
7. the mean number of patient-days spent on a mechanical ventilator;
8. the median of total number of patient-days spent on a mechanical ventilator;
9. the mean length of hospital stay in days of ill/injured survivors; and
10. the median length of hospital stay of ill/injured survivors.

Mortality rates are indicators of the magnitude of the disasters and its overall burden on the community. Rates of deaths, also termed mortality rates, may be more indicative than absolute numbers of deaths^116^. Several factors are likely to affect mortality rates in disasters, including hazard characteristics which in turn will affect illness/injury type and severity, the timeliness of the medical response, the casualty load, and the capacity and quality of medical care on the scene, in the ED and in the healthcare facility^116^.

The time interval between the onset of the illness or injury to definitive care is an important prognostic factor that can affect survivor outcome^29^ ^161^. Reports from past disasters have indicated a direct correlation between the time interval from rescue to definitive care and survivor mortality^29^ ^126^ ^141^ ^161^ ^192^ ^193^ ^194^ ^195^.

In assessing the effectiveness of DMR, we need to distinguish between the immediate deaths due to the hazard impact and the number of deaths that could have been potentially prevented, if ill/injured survivors were provided with timely medical attention, e.g. pre-hospital deaths, in-hospital deaths and “immediate” category deaths^67^. The overall mortality rate in disasters is skewed by the large number of the non-severely injured. The mortality rate of the “immediate category” survivors may be a more useful indicator of the effectiveness of the DMR, and allows a more meaningful comparison of outcomes from different disasters^67^ ^196^. The Group suggests that further research efforts should focus on the impact of medical, organizational, structural and cultural differences among disaster medical response systems on the morbidity and mortality of disaster survivors.

The Group proposes the following indicators for measuring mortality:

1. the percentage of impact deaths within the total population of disaster victims;
2. the percentage of pre-hospital deaths within the total population of disaster victims;
3. the percentage of in-hospital deaths within the total population of disaster victims;
4. the percentage of “immediate category” deaths within the total population of “immediate category” survivors.

## General Discussion

Similar to other branches of medicine, disaster medicine must possess a scientific basis. To date, there is no evidence-based literature that clearly defines the best medical response principles, concepts, structure and processes in the disaster setting^7^ ^191^ ^197^. The DMR is only as good as the assumptions on which it is based. Many of these assumptions are incorrect and/or are not based on systematically collected evidence^102^ ^103^ ^198^. Much of what is currently known about DMR results from descriptive studies and expert opinion^7^ ^102^. These insights are important, but lack the impact and the extrapolation power of conclusions drawn from objective data. It is not always easy to draw general conclusions based on single-centre studies, as different outcomes have been used, inclusion criteria vary, and structural, organizational and cultural differences among medical response systems may further hinder this process^199^. However, there is an increasing awareness among the speciality for the need to collect data that supports valid, reproducible conclusions on the effectiveness of the DMR^184^.

A prerequisite to adopting any evidence-based approach to DMR is the need to assemble a body of evidence from the results of relevant empirical studies^13^ ^197^. In situations of emergency, the gathering of reliable and valid data will always be difficult, as most healthcare providers prioritize the deliverance of care to a large number of victims over the documentation of cases during this time^4^ ^102^ ^104^. Furthermore, much of the relevant data becomes less accessible over time. Thus, it is important to establish dedicated field research teams that can be mobilized quickly after the onset of the disaster. Much of the data published worldwide is found in non–peer-reviewed publications, in unpublished reports or appears in the “grey” literature of government agencies or academic institutions. These are difficult to access, particularly through electronic indexing services^102^.

Health professionals and researchers from a broad spectrum of disciplines and specialities, contribute to the knowledge base of disaster medicine science. Although multidisciplinarity can be a strength, it can also be a hindrance due to differences of training and professional backgrounds. Moreover, there is a large variety in the organization of the DMR, both within and between countries. Without a standardized framework for describing and reporting the features that impact DMR, it is very difficult to compare results of DMR evaluations, and even more difficult to identify best practices^2^ ^5^ ^6^ ^7^ ^8^. The lack of a common language also hinders intra- and interdisciplinary collaborations and appropriate training. Although there is seldom full agreement on all concepts in a particular field, it is counterproductive to have multiple, inconsistent and contradictory usages of many key terms. Unfortunately we found that this would describe the current situation in the disaster medicine discipline.

It is recognized that the study of DMR is a relatively new field. Developing a set of data elements, characterizing the medical response, and their indicators, based on research evidence, is in early stages. The identification and use of relevant indicators is a crucial part of determining the impact of interventions in DMR. The literature generally distinguishes between process or performance indicators and impact or outcome indicators. Performance indicators concern both the output of activities or interventions conducted and the process of implementation of the interventions. Outcome indicators are measures of the actual achievements intended, in our case, by the DMR. Mortality and morbidity rates are the most commonly used outcome indicators for medical management 1 15. The existing indicators, which have emerged through professional consensus, are not generally accepted and agreed on^7^. Without indicators that are measuring a DMR in a reliable and valid manner, it will not be possible to perform comparisons across different DMR systems, nor to evaluate the impact of response interventions, or to analyse the results in a scientific way. The Group trusts that the development of the indicators included in this Utstein-style template will serve as a framework for assessing the effectiveness of the DMR in the future. Moreover, collection of consistent data could lead, over time, to the development of a consensus on, and validation of, evidence-based process and outcome indicators.

The Group is mindful of the potential implementation difficulties that relate to Utstein-style templates^11^ ^200^ ^201^ . To address this concern, the template developed in this project will be made widely available by dissemination by the EMDM Academy, the research centres affiliated with the EMDM Academy, and the EMDM Alumni Association. To further facilitate implementation, a complete user manual and online template will be available for download free of charge at the respective websites.

The EMDM Academy Consensus Group encourages all readers to assist in the initial testing and immediate use of the template. Journal editors will be encouraged to adopt this Utstein-style template as reporting requirements for studies on DMR. These requirements will ensure consistent reporting of data and will allow reviewers and readers to accurately evaluate a study^7^.

Notwithstanding the fact that the vast majority of disasters occur in developing countries, peer reviewed publications on the effectiveness of the medical response to disasters in these countries are rare or not readily available^202^. Moreover, it is difficult to determine how applicable are research findings regarding DMR management, derived mostly from developed countries, to developing societies^203^. The Group hopes that this Utstein-style template for uniform reporting of acute DMR will encourage DMR research studies in developing countries.

The template needs to be field- and pilot-tested. Before formal pilot-testing, the template will require a more formative field-testing process. Such field tests are targeting the feasibility of using the tool and will provide feedback on the types of revisions that will improve its usability. Although the Utstein-style template can be used more easily for discrete scene disasters, it can be employed in large-scale events by collecting the data for each sector of the widespread scene. Similarly, data should be gathered for each individual healthcare facility and subsequently compiled, as appropriate. Formal pilot-testing would involve a greater number of disasters in order to provide a robust picture of the template’s performance across diverse types and scales of disasters. Currently, two studies are applying the template by using historical data from previous disasters and any modifications that will be deemed necessary will be incorporated as appropriate. The template is a dynamic document and will no doubt undergo a number of revisions as more data and experiences accumulate^200^. A follow-up meeting within two years will be held in order to revise the template and incorporate field studies, the experiences obtained with the first template, and any pertinent new research. However, the planned revision should not preclude the implementation of the current version of the template.

Databases available for DMR research are underdeveloped, incomplete and inaccurate^13^ ^204^ ^205^ . Although efforts have been made after the Asian tsunami to collect evidence by systematic reviews on a number of medical treatment interventions, no such studies have been performed concerning the effectiveness of disaster medical system interventions on the health outcomes of disaster survivors^13^ ^206^ . A uniform reporting method and template are essential in order to collect empirical data on DMR management. Such robust databases will allow DMR investigators to collect and interpret valid evidence that will impact disaster survivors’ outcomes^13^ . The EMDM Academy will provide the database infrastructure for the purpose of collecting, collating, and analysing data with respect to DMR and for facilitating multi-institutional research efforts in a standardized data set. Furthermore, individual data from disaster victims will also be collected in a specific database, i.e. VictimBase, an online victim library for medical professionals to create and share disaster victim profiles^207^ . Additionally, disaster victims from existing trauma or hospital registries should be earmarked so that data can easily be collected.

## Conclusion

The present Utstein-style template is compiled by the EMDM Academy Consensus Group comprising of international representatives and provides a set of data elements for uniform reporting on the acute DMR. This will encourage more complete data collection as well as consistency in the reporting of findings and will enable comparison of research studies on medical response systems, both nationally and internationally. The data elements, definitions and indicators relating to the acute DMR identified in the present report will permit the collection of the most meaningful data, aggregate data analyses, and determination of the impact of different medical response strategies on outcome. This may contribute to further scientific evidence and knowledge related to acute DMR in order to optimize system interventions and to improve clinical outcomes of disaster survivors. This study on uniform reporting of acute medical response in disasters should be completed by data elements and indicators measuring the public health interventions and the mental health support.

## Competing Interests

No competing interests exist.

## Appendix A

### Medical Operation Coordination Scale

**Table:**

**Item**	**Score**							**Notes**
**1.On-scene Initial Actions **								
First medical responder reports arrival on scene	0	1	2	3	4	N/A	N/D	
First medical responder assumes role of medical incident commander	0	1	2	3	4	N/A	N/D	
Protection of responders and victims	0	1	2	3	4	N/A	N/D	
Preliminary assessment of the incident by first medical responder	0	1	2	3	4	N/A	N/D	
First situation report to dispatch centre or equivalent by first medical responder	0	1	2	3	4	N/A	N/D	
Request for appropriate additional personnel and non-personnel resources	0	1	2	3	4	N/A	N/D	
Assignment of initial response roles on the scene of the incident	0	1	2	3	4	N/A	N/D	
	**Total score**							
**2.On-scene Medical Control and Coordination**								
Designated Incident Medical Commander (IMC) reports arrival at scene	0	1	2	3	4	N/A	N/D	
Report of temporary IMC (first responder) to designated IMC	0	1	2	3	4	N/A	N/D	
Establishing and operating of site-level medical command post								
Medical assessment of the incident by IMC	0	1	2	3	4	N/A	N/D	
Determining numbers/skill mix of personnel needed for optimal response to the incident (above and beyond those currently deployed)	0	1	2	3	4	N/A	N/D	
Determining numbers/skill mix of personnel currently available for deployment	0	1	2	3	4	N/A	N/D	
Determining non-personnel resources needed for optimal response to the incident (above and beyond those currently deployed)	0	1	2	3	4	N/A	N/D	
Determining non-personnel resources currently available for deployment	0	1	2	3	4	N/A	N/D	
Medical situation report(s) from IMC to Incident Commander	0	1	2	3	4	N/A	N/D	
Medical situation report(s) from IMC to medical representative in Disaster Operational Centre (s)	0	1	2	3	4	N/A	N/D	
Medical situation reports from IMC to medical healthcare facilities	0	1	2	3	4	N/A	N/D	
Selection by IMC of areas to establish triage, treatment, distribution and transportation of ill/injured survivors, and a temporary morgue	0	1	2	3	4	N/A	N/D	
Assignment by IMC of ancillary functions for medical response (search and rescue, triage, treatment, patient distribution, transportation, medical logistics, communications, morgue)	0	1	2	3	4	N/A	N/D	
Establishment of an incident medical action plan by IMC	0	1	2	3	4	N/A	N/D	
Situation reports from ancillary functions to IMC regarding status of incident	0	1	2	3	4	N/A	N/D	
IMC briefing(s) to update medical command staff	0	1	2	3	4	N/A	N/D	
Establishment of proper record keeping	0	1	2	3	4	N/A	N/D	
Termination of medical field operations	0	1	2	3	4	N/A	N/D	
	**Total score**							
**3.System-level Medical Coordination**								
Notification of the assigned healthcare organization commander (EMS, hospitals, …)	0	1	2	3	4	N/A	N/D	
Reporting of the assigned healthcare organization commander at appropriate location	0	1	2	3	4	N/A	N/D	
Notification of the assigned on-scene medical commander	0	1	2	3	4	N/A	N/D	
Reporting of the assigned medical commander at the incident command post	0	1	2	3	4	N/A	N/D	
Notification of the assigned medical staff at local coordination centre	0	1	2	3	4	N/A	N/D	
Reporting of designated medical staff at local coordination centre	0	1	2	3	4	N/A	N/D	
Notification of the assigned medical staff at regional coordination centre	0	1	2	3	4	N/A	N/D	
Reporting of the assigned medical staff at regional coordination centre	0	1	2	3	4	N/A	N/D	
Notification of the assigned medical staff at federal coordination centre	0	1	2	3	4	N/A	N/D	
Reporting of the assigned medical staff at federal coordination centre	0	1	2	3	4	N/A	N/D	
Information flow between the different medical coordination tiers	0	1	2	3	4	N/A	N/D	
Conducting operational after-action debriefing	0	1	2	3	4	N/A	N/D	
	** Total score**							
**4. Medical Communications and Information Management**								
Identification of intra- and interjurisdictional health/medical stakeholders to be incorporated into the information flow	0	1	2	3	4	N/A	N/D	
Establishment of communication pathways between (medical) command, management locations and support agencies	0	1	2	3	4	N/A	N/D	
Maintenance of continual connection with all parties involved throughout the incident management	0	1	2	3	4	N/A	N/D	
Maintaining situational awareness using information gathered from medical stakeholders	0	1	2	3	4	N/A	N/D	
Establishment of patient tracking procedures	0	1	2	3	4	N/A	N/D	
Development and dissemination of information, alert or warnings to the public	0	1	2	3	4	N/A	N/D	
Development and dissemination of information, alert, warnings or notifications to the incident managers and responders	0	1	2	3	4	N/A	N/D	
	** Total score**							
**5.Medical Resource Management**								
Mobilization of EMS healthcare personnel	0	1	2	3	4	N/A	N/D	
Mobilization of medical facilities personnel	0	1	2	3	4	N/A	N/D	
Mobilization of medical transportation means	0	1	2	3	4	N/A	N/D	
Mobilization of medical equipment	0	1	2	3	4	N/A	N/D	
Mobilization of medical supplies	0	1	2	3	4	N/A	N/D	
Establishment of alternate care facilities	0	1	2	3	4	N/A	N/D	
Assessment of resource requirements based on the evolving situation	0	1	2	3	4	N/A	N/D	
Demobilization of resources	0	1	2	3	4	N/A	N/D	
	** Total score**							

0 – Should have been done, but was not.

1 – Inadequate. The action or activity was started but was so incomplete or poorly performed as to be ineffective.

      Did not function

2 – Somewhat adequate. The action or activity was started and was partially complete and/or performed in a partially effective manner.

      Did function, but needs major improvement.

3 – Mostly adequate. The action or activity was performed in a generally effective and complete manner.

      Did function, needs minor improvement.

4 – Completely adequate. The action or activity was completely performed in a fully effective manner.

      Did function well.

N/A – Not applicable. The action or activity was not appropriate or necessary for this operation.

N/D – Not able to determine.
